# Reporting of Magnetic Resonance Enterography in Inflammatory Bowel Disease: Results of an Italian Survey

**DOI:** 10.3390/jcm13133953

**Published:** 2024-07-05

**Authors:** Cristiana Bonifacio, Arianna Dal Buono, Riccardo Levi, Roberto Gabbiadini, Christian Reca, Cristina Bezzio, Marco Francone, Alessandro Armuzzi, Luca Balzarini

**Affiliations:** 1 Department of Radiology, Humanitas Research Hospital—IRCCS, Via Manzoni 56, Rozzano, 20089 Milan, Italy; cristiana.bonifacio@humanitas.it (C.B.); riccardo.levi@humanitas.it (R.L.); christian.reca@humanitas.it (C.R.); marco.francone@hunimed.eu (M.F.); luca.balzarini@humanitas.it (L.B.); 2 IBD Center, Humanitas Research Hospital—IRCCS, Via Manzoni 56, Rozzano, 20089 Milan, Italy; roberto.gabbiadini@humanitas.it (R.G.); cristina.bezzio@hunimed.eu (C.B.); alessandro.armuzzi@hunimed.eu (A.A.); 3 Department of Biomedical Sciences, Humanitas University, Pieve Emanuele, 20072 Milan, Italy

**Keywords:** reporting, cross-sectional imaging, magnetic enterography, inflammatory bowel disease, survey

## Abstract

**Background/Objectives:** Inflammatory bowel diseases (IBDs) are chronic disorders that require close monitoring with imaging techniques such as magnetic resonance enterography (MRE). Standardization of radiological reports is crucial for the optimal management of IBD. We surveyed Italian radiologists regarding their experiences with MRE examinations and reporting for IBD. **Methods:** All members of the Italian Society of Medical and Interventional Radiology (SIRM) were invited to complete an anonymous questionnaire in April 2023. Comparison tests between variables were assessed using the χ^2^ test or Fisher exact test according to the least frequency group. Significance level was set for *p*-value < 0.05. **Results:** A total of 253 radiologists responded to the survey. Around 70% of the respondents declared personal clinical experience with IBD. Great agreement with the items included and described for both disease activity (i.e., intestinal wall thickness, presence of mucosal ulcers, presence of edema, mucous enhancement) and complications was reported. One-third of the respondents regularly used a structured MRE report. Centers with a high number of IBD patients per year (>1000) mostly used 3 T scanners or both 1.5 T and 3 T scanners (*p* < 0.001). The incorporation of scores of disease activity was associated with university and high-volume hospitals (*p* < 0.001). **Conclusions:** This survey highlighted the current routine practice and experience of MRE reports of IBD patients among Italian radiologists. We found deficiencies in the use of radiological scores in MRE reports and attendance at IBD multidisciplinary meetings.

## 1. Introduction

Inflammatory bowel diseases (IBDs) are chronic, disabling, and incurable conditions of the gastrointestinal tract that exhibit a relapsing–remitting behavior [1]. The burden of IBDs is rising globally, and the prevalence in Europe and the United States is estimated at 1 in every 200–300 people [2]. 

Close monitoring and adequate assessment of response to therapy through objective measures are currently the backbone of managing IBDs [3,4]. Cross-sectional imaging methods, including intestinal ultrasound (IUS), magnetic resonance enterography (MRE), and computed tomography (CT), have been demonstrated as being highly accurate for the diagnosis and assessment of disease activity and severity, and for detecting complications in IBDs [5,6]. The pooled sensitivity and specificity of cross-sectional imaging are estimated to be >85% and >95%, respectively, with few differences among the techniques regarding disease extension, location, severity, and complications [7].

Several studies have demonstrated that the achievement of transmural healing, alongside with mucosal healing, is a complementary predictor of better long-term clinical outcomes in CD (i.e., lower risk of hospitalization, surgery, and/or corticosteroid use) [8,9,10]. Indeed, transmural healing, evaluated through MRI and defined as a bowel thickness <3 mm, without an increased T2 mural intensity or increased contrast enhancement on T1 sequences, was associated with reduced hospitalization rates, surgery, and treatment escalation over 5 years of follow-up in CD patients [8].

At present, the regular assessment of radiological response is recommended after therapy starts, with modification and/or escalation in IBD patients, especially in CD [4].

Recently, both gastroenterological and radiological societies have published guidelines that establish technical standards for cross-sectional imaging in IBD, define parameters, and indicate how to report the results [11,12,13]. Standardization of radiological reports is crucial for the optimal management of IBD in order to reliably guide therapeutic decision making. At present, there are no national guidelines regarding radiological monitoring in patients with IBD and the behavior of Italian Centers towards MRE in IBD patients is unknown. The aim of this study was to investigate technical and reporting standards in the use of MRE among Italian radiologists involved in the management of IBD patients. We evaluated radiologists’ experiences with MRE examinations for IBD and with the reporting of MRE results by surveying members of the Italian Society of Medical and Interventional Radiology (SIRM).

## 2. Materials and Methods

To explore the application of MRE in the assessment of IBD patients by Italian radiologists, we developed an anonymous survey that was endorsed by SIRM. The questionnaire was developed using a dedicated software platform (Google forms, Google, Mountain View, CA, USA) and was anonymous. 

### 2.1. Participants

In April 2023, a sample of SIRM members received an email invitation to complete the survey (*n* = 934). The survey remained available for 14 consecutive days, and participants could access it only once. A reminder was sent 4 days before the survey’s closure. All completed questionnaires were included in the analysis.

### 2.2. Procedures

To estimate the time needed to complete the survey (approximately 6–8 min), we asked 10 colleagues to time themselves on a test run before opening the survey to SIRM members. 

The survey comprised 21 multiple choice questions, of which 14 permitted only one answer and 7 permitted more than one. The questionnaire was administered in Italian, while an English translation is provided in Table 1. 

Overall, 10 questions inquired about the respondents’ demographics (age, professional qualifications, experience in MRE for IBD) and work setting, whereas 11 questions focused on the use of MRE for IBD (i.e., machines and protocols) and the reporting of these results (i.e., items assessed and described in the radiological report for disease activity, severity, and complications) (Table 1). 

Statistical analyses were performed on survey answers as categorical data. Data were described as absolute count and relative percentages. Comparison tests between variables were assessed using the χ^2^ test or Fisher exact test according to the least frequency group. A significance level was set for *p*-value < 0.05.

## 3. Results

### 3.1. General Questions, Experience in IBD, Characteristics of the Centers

A total of 253 radiologists responded to the survey (response rate 21.7% of the invited), and 45.3% of respondents had an age between 35 and 50 years (*n* = 115). The study group comprised 191 radiologists (75.5%) and 62 radiology residents (24.5%). Just under half of respondents (*n* = 119; 47.1%) worked in a public hospital, while 92 (36.3%) worked at a university hospital and 42 (16.6%) worked at a private hospital or clinic. The respondents worked across Italy, and 188 (74.3%) claimed to have experience with IBD. Table 2 elucidates the main characteristics of the study population. 

In terms of the participants’ place of work, 97 respondents (38.3%) worked in a setting with an IBD case load below 100 patients/year, and 80 respondents (31.6%) reported a case load between 100 and 500 patients/year, whereas 76 respondents (30.1%) reported a case load above 500 patients/year. Regarding the clinical management and discussion of imaging findings, 121 respondents (47.8%) indicated having a multidisciplinary IBD meeting in their hospital, with a higher presence in university hospitals compared with public hospitals: 76.1% and 30.0%, respectively (*p* < 0.001). 

MRE examinations were mostly performed on 1.5 T MRI scanners (*n* = 189, 74.7%), while small numbers of respondents indicated that they used both 1.5 and 3 T scanners (38/253, 15.0%) or only 3 T scanners (26/253, 10.3%). The use of 1.5 T and 3 T scanners was found to be different among Centers with a higher percentage of 1.5 T in public (87.5%) and non-hospital Centers (87.5%) (*p* < 0.001), whereas 3 T scanners were mostly used in private hospitals (23.5%) and university hospitals (12.0%) (*p* < 0.001). Notably, Centers with high numbers of patients with IBD per year (>1000) were mostly associated with 3 T scanners (17.2%) or both 1.5 T and 3 T scanners (48.3%) (*p* < 0.001).

**Figure 1:**
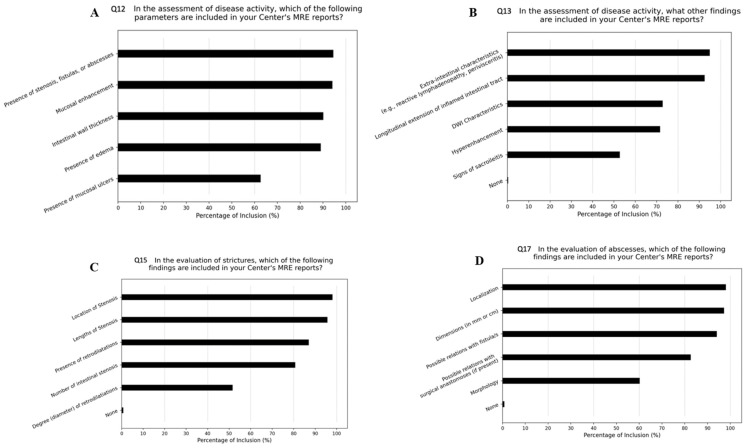
Details of answers to questions regarding assessment of disease activity and complications.

Figure 1 summarizes the answers to questions regarding the assessment of disease activity and complications.

In Figure 1, the main items investigated through the questions are about disease activity and complications. In Q12 (panel A), the general features of transmural activity were asked to the participants (i.e., intestinal wall thickness, mucosal enhancement, mucosal ulcers and/or complications); Q13 (panel B) addressed adjunctive radiological features of active disease (i.e., extra-intestinal characteristics, concomitant sacro-ileitis); and Q15 and Q17 (panels C and D, respectively) specifically addressed items included for describing strictures and abscess/es.

### 3.2. Reporting Details

The second part of the questionnaire focused on MRE examinations and reports. When asked if the results of MRE examinations for patients with IBD were reported on a structured radiological report, only 77 respondents answered yes (30.4%). No significant association was found between the use of structured forms and the geographical area of Italy (*p* = 0.553), type of hospital (*p* = 0.606), or professional qualification (*p* = 0.442). Even fewer respondents indicated that they routinely include IBD clinical scores in the reports (*n* = 26, 10.3%) or include a score only when requested by a gastroenterologist (*n* = 39, 15.4%). The use of IBD activity scores was highest among respondents based in Northern Italy (76.9% vs. 23.1% for Central Italy, Southern Italy and islands, *p* = 0.004). It was also higher for radiology residents working at university hospitals (57.7% vs. 42.3% for radiologists, *p* = 0.005). 

Concerning the evaluation of IBD activity (Figure 1A), half of respondents (*n* = 138, 54.5%) reported including all the following items: intestinal wall thickness, presence of mucosal ulcers, presence of edema, mucous enhancement. Moreover, almost all respondents include the presence of fistulas or abscesses (*n* = 240, 94.9%). Approximately one-third of the respondents (*n* = 77, 30.4%) include further descriptive items in the MRE report (Figure 1B), such as extra-intestinal features (i.e., reactive lymphadenopathy, perivisceritis), hyperenhancement, DWI features, the longitudinal extent of the inflamed intestinal tract and signs of sacro-ileitis.

In the evaluation of IBD-related stenosis (Figure 1C), more than 86.5% (*n* = 219) of the respondents include a numerical evaluation of wall thickness (in mm). More than one-third of the respondents (*n* = 109, 43.1%) consider it valuable to include the following items in the radiology report: the number of intestinal strictures, the location of the stricture(s), the length of the stricture(s), the presence and the degree (diameter) of pre-stenotic dilation. Moreover, 179 (70.7%) of the respondents found it relevant to report the following findings related to stenosis in the MRE report: the relationship to surgical anastomoses (if present), the presence of signs of active inflammation within the stricture, and the presence of fistulas/abscesses adjacent to the stricture.

Concerning the evaluation of abscesses (Figure 1D), 56.1% (*n* = 142) included all the following findings: location, dimensions (in mm or cm), morphology, and any relationship with fistula(s) and with surgical anastomoses (if any). The most frequently included feature in the report was the location of the abscess (*n* = 249, 98.4%).

### 3.3. Structured Report, Scores of Disease Activity, and Multidisciplinary Meetings

**Figure 2:**
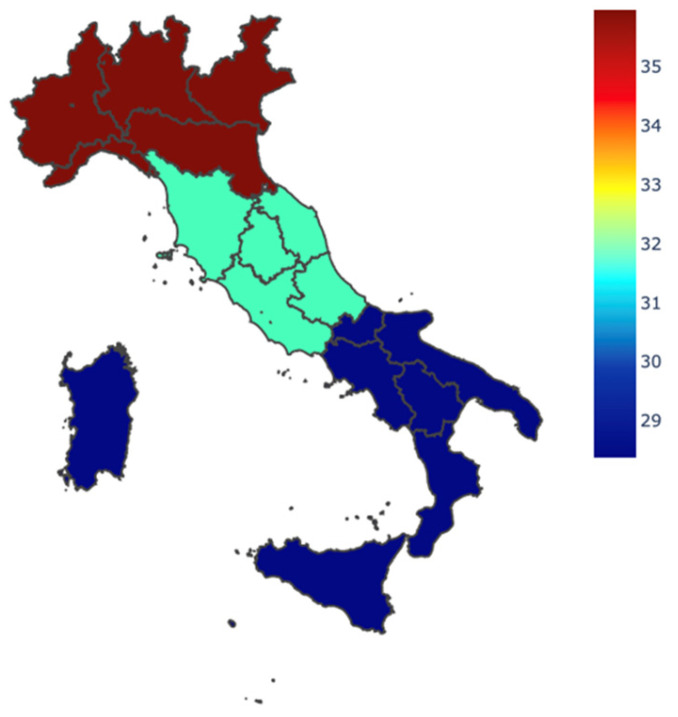
Percentage of usage of structured report for patients with IBD among geographical areas.

Overall, a structured MRE report was found to be used by a small percentage of respondents (*n* = 83, 32.8%). In more detail, 58 respondents (22.9% of the overall study population, 68.8% of users) said they use it on a daily basis, whereas the remaining 25 respondents use it only for clinical trials or upon gastroenterological request. Among the respondents, no differences were found in the use of a structured report in relation to geographical location (*p* = 0.553) (Figure 2), working qualification (*p* = 0.442), or type of Center (*p* = 0.606).

Regarding the adoption of scores in the MRE report, those sampled routinely include IBD clinical scores in reports (*n* = 26, 10.3%) in their daily practice: the use of IBD activity score/s in MRE reports is mainly adopted in the north of Italy (17.5%, *p* = 0.004), by radiology residents (25.4%, *p* < 0.001), and in university hospital (38.4%, *p* < 0.001) (Table 3).

The most adopted score is the simplified MaRIA score (*n* = 12/65, 46.2%), followed by the MaRIA score (*n* = 18/65, 27.7%) and MEGS (*n* = 1/65, 4%) (Table 3). 

High confidence in MaRIA/sMaRIA score use was reported by only 8 respondents (30.7% of users, 3.1% of all respondents), and moderate confidence was reported by 53 respondents (81.5% of users, 20.9% of all respondents) (Table 3).

Multidisciplinary IBD meetings are accessible for 121 respondents (47.6%) (Table 2). Among those, 25 radiologists reported always participating (20.7%), 29 participate only for complex cases (24.0%), 43 are interested but not invited (35.5%), and 24 are not interested in participating (19.8%) (Table 2). Italian Centers in central regions (18,2%, *p* = 0.014), respondents between ages 35 and 50 years (13.0%, *p* < 0.001), and those working in university hospitals (18.5%, *p* < 0.001) were associated with higher participation in multidisciplinary IBD meetings.

## 4. Discussion

The increased availability and the accuracy of cross-sectional imaging in detecting complications, monitoring therapeutic response, and predicting long-term clinical outcomes place the radiologist at center stage in the management of IBD [14].

Our survey sought to highlight the current routine practice and experience with MRE reports for patients with IBD among Italian radiologists, highlighting high and low points. To the best of our knowledge, this is the first survey addressed to all members of a national Italian radiological society on this topic. This survey was mostly completed by radiologists with a declared personal expertise in IBD (>70% of the participants), thus representing a close picture of the daily clinical practice in this field.

An important result of the survey is the great agreement with the items included and described for both disease activity and complications (around 55% and 86–94% of agreement, respectively). This result lays the foundations for homogeneous and optimal MRE reporting among Italian radiologist with experience in IBD and can improve communication between different specialties. On the other hand, only 22.9% of the respondents use a structured reports in their daily practice, and most of them scarcely adopt radiological scores, with very low rates of confidence only concerning the MaRIA/sMaRIA scores. This is a relevant drawback, since it has been demonstrated that the MaRIA score is highly effective in detecting mucosal healing in CD [15,16], supporting its use as a therapeutic endpoint and maybe reducing the number of colonoscopies requested for monitoring patients. Calculating the MaRIA score can be time-consuming; however, the simplified MaRIA, which requires less time (sMaRIA: median 4.50 min vs. MaRIA: median 12.35 min), was thereafter developed to overcome this possible limitation [17,18] and can assess CD activity with a high correlation with the simple endoscopic score for CD (SES-CD) and excellent inter-rater reliability between expert and resident radiologists [6,19]. Accordingly, our survey showed that the most used score is the sMaRIA, and the adoption of scores was found to be most frequent among radiology residents (40.7%, *p* = 0.005), in university hospitals (38.0%, *p* = 0.004), and in hospitals with more than 1000 patients with IBD per year (55.2%, *p* < 0.001).

Another main issue of our study regards the involvement of radiologists in multidisciplinary IBD meetings, which appears to be higher in university hospitals compared with public hospitals (76.1% and 30.0%, respectively (*p* < 0.001)). This topic should be stressed and implemented in clinical practice as it seems that, similarly to oncology, multidisciplinary meetings can ameliorate the clinical management of IBD and represent a standard quality of care as expressed by the European Crohn’s and Colitis Organisation and European Society of Radiology [20,21,22].

Consistently with previous data [23], MRE is performed with both 1.5 T and 3 T scanners, with a wider accessibility with 1.5 T. Few data are available on the performance differences between the two scanners in IBD, showing a slight superiority of 3 T as concerns the detection of mucosal ulcers [24]. In addition, 3 T displayed a shorter scan time. Therefore, if at one’s disposal, 3 T is preferred in patients with ileo-colonic CD [24]. However, larger studies are warranted to better assess this issue.

Our study has some limitations. First, despite the declared experience with IBD of the participants, few highly specialized/third-level Centers were included (approximately 30% of high-volume IBD Centers [>500 patients treated yearly]). The members of the SIRM currently number approximately 12,000, and the participants in this survey are evidently radiologists with a specific interest in the field of IBD, with a possible associated selection bias. Secondly, few questions addressed technical aspects regarding MRE protocol. Finally, this survey did not separate pediatric from adult physicians, with possible mixed results.

Overall, this study helps to determine and understand the differences that exist in IBD diagnostic strategies between Italian Centers and radiologists, emphasizing common and widespread conduct, as well as areas for possible growth and improvement.

## 5. Conclusions

Our study helps provide an understanding of the current reporting standards among Italian radiologists specifically regarding MRE in patients with IBD. These data might improve radiology practices with respect to the incorporation of relevant items and descriptions to drive medical decisions more accurately, finally improving the outcomes of patients with IBD. According to our results, investing resources in dedicated educational and training programs in this field appears to be of evident importance. These findings can be used for better standardization of the reporting in MRE, specifically regarding the use of a structured report and the incorporation of MRE scores of disease activity, thus improving the quality of patients’ care.

## Figures and Tables

**Table 1:** Questions and items included in the administered questionnaire.

Q1	Where do you work?	Northern Italy Center Italy Southern Italy and islands
Q2	How old are you?	<35 years 35–50 years >50 years
Q3	What is your qualification?	Hospital doctor Resident Independent practitioner
Q4	In what type of facility do you work?	Public Hospital University Hospital Private Hospital/Clinic Non Hospital structure
Q5	How many patients with IBD are treated at your Center annually?	<100 100–500 500–1000 >1000
Q6	How many MRE are performed at your Center weekly (only for patients with IBD)?	≤1 2–3 4–5 >5
Q7	Which magnetic field is used in your Center for enteric MRI?	1.5 Tesla 3 Tesla 1.5 and 3 Tesla
Q8	Do you have experience in IBD?	Yes No
Q9	If yes, how many years have you been involved in IBD?	<5 years 5–10 years >10 years
Q10	Do you usually use a structured report for MRE of patients with IBD?	Yes Upon request Only for clinical trials No
Q11	Among general findings, what is included in your Center’s reports for MRE of patients with IBD (multiple answers possible)?	Clinical indications Technical details Oral contrast volume IBD clinical characteristics None of the above
Q12	In the assessment of disease activity, which of the following parameters are included in your Center in the reports of MRE of patients with IBD (multiple answers possible)?	Presence of stenosis, fistulas or abscesses Mucosal enhancement Intestinal wall thickness Presence of edema Presence of mucosal ulcers
Q13	In the assessment of disease activity, what other findings are included in your Center in the reports of MRE of patients with IBD (multiple answers possible)?	Extra-intestinal characteristics (e.g., reactive lymphadenopathy, perivisceritis) Longitudinal extension of inflamed intestinal tract DWI characteristics Hyperenhancement Signs of sacroiliitis None of the above
Q14	Do you usually quantify numerically the wall thickness (in mm)?	Yes Upon request No
Q15	In the evaluation of stenosis, which of the following findings are included in your Center in the reports of MRE of patients with IBD (multiple answers possible)?	Location of stenosis Lengths of stenosis Presence of pre-stenotic dilation Number of intestinal stenoses Degree (diameter) of pre-stenotic dilation None of the above
Q16	In the evaluation of stenosis, which of the following findings are included in the reports of MRE of patients with IBD at your Center (multiple answers possible)?	Presence of adjacent fistulas/abscesses to the stenosis Presence of signs of active inflammation within the stenosis Relation to surgical anastomosis (if present) None of the above
Q17	In the evaluation of abscesses, which of the following findings are included in the reports of MRE of patients with IBD at your Center (multiple answers possible)?	Localization Dimensions (in mm or cm) Possible relations with fistula/s Possible relations with surgical anastomoses (if present) Morphology None of the above
Q18	Do you use scores in the assessment of IBD at your Center?	Yes, at least one Only upon gastroenterologist’s request No
Q19	If yes, which scores are usually included (multiple possible)?	MaRIA score MaRIA simplified score MEGS score
Q20	How confident do you feel with MaRIA or MaRIAs?	Very confident Rather confident Non confident
Q21	Do you participate as a radiologist in multidisciplinary IBD meetings?	Always Only for complex cases and if invited by colleagues I’m interested, but I’m not involved I’m not interested Multidisciplinary IBD meetings is not present in my center

IBD: inflammatory bowel diseases; MRE: magnetic enterography; MaRIA: Magnetic Resonance Index of Activity; MEGS: magnetic resonance global score.

**Table 2:** Characteristics of the radiologists who responded to the questionnaire and of their work settings.

Characteristics		*n* = 253 *n* (%)
Age class		
	<35 years	79 (31.1)
	35–50 years	115 (45.3)
	>50 years	59 (23.6)
Professional qualification		
	Radiologist Hospital doctor Independent practitioner	191 (75.5) 166 (86.9) 25 (13.1)
	Radiology resident	62 (24.5)
Site of employment		
	Public hospital	119 (47.1)
	University hospital	92 (36.3)
	Private hospital or clinic	34 (13.4)
	Non-hospital structure	8 (3.2)
Geographic area of work		
	Northern Italy	113 (44.9)
	Central Italy	66 (26.0)
	Southern Italy or islands	74 (31.1)
Experience with IBD		
	Yes	188 (74.3)
	No	65 (25.7)
Duration of IBD experience, years		
	<5	68 (36.2)
	5–10	59 (31.4)
	>10	61 (32.4)
Hospital case load of patients with IBD, number per year		
	<100	97 (38.3)
	100–500	80 (31.6)
	500–1000	47 (18.6)
	>1000	29 (11.5)
Hospital case load of MRE examinations for IBD, number per week		
	≤1	74 (29.2)
	2–3	95 (37.6)
	4–5	35 (13.8)
	>5	49 (19.4)
Participation in multidisciplinary IBD meetings		
	Yes, always	25 (9.9)
	Mainly for complex cases and when invited by colleagues	29 (11.5)
	I’m interested, but I’m not involved	43 (17.0)
	I’m not interested in participating, although there is a multidisciplinary IBD meeting in my center	24 (9.5)
	No, because multidisciplinary IBD meetings are not held in my center	132 (52.1)
MRI scanner’s magnetic field		
	1.5 Tesla	189 (74.7)
	3 Tesla	26 (10.3)
	1.5 and 3 Tesla	38 (15.0)

MRI: magnetic resonance imaging; IBD: inflammatory bowel disease.

**Table 3:** Statistical results for structured MRE report and disease activity scores.

	Adoption of Structured MRE Report	Adoption of Disease Activity Scores
Geographical Location	χ^2^ = 1.20, *p*-value = 0.55 ^1^	χ^2^ = 15.28, *p*-value = 0.004 ^1^
Responder’s Age	χ^2^ = 3.17, *p*-value = 0.21 ^1^	χ^2^ = 15.70, *p*-value = 0.003 ^1^
Type of Hospital	*p*-value = 0.606 ^2^	*p*-value = 0.005 ^2^
Working Occupation	*p*-value = 0.442 ^2^	*p*-value = 0.004 ^2^

^1^ Chi-squared test; ^2^ Fisher exact test.

## Data Availability

The datasets generated or analyzed during the study are available from the corresponding author on reasonable request.
